# Identification of host proteins interacting with the integrin-like A domain of *Toxoplasma gondii* micronemal protein MIC2 by yeast-two-hybrid screening

**DOI:** 10.1186/s13071-014-0543-1

**Published:** 2014-11-26

**Authors:** Yifan Wang, Rui Fang, Yuan Yuan, Min Hu, Yanqin Zhou, Junlong Zhao

**Affiliations:** State Key Laboratory of Agricultural Microbiology, College of Veterinary Medicine, Huazhong Agricultural University, Wuhan, 430070 Hubei PR China; Key Laboratory of development of veterinary diagnostic products, Ministry of Agriculture, Huazhong Agricultural University, Wuhan, 430070 Hubei PR China

**Keywords:** *Toxoplasma gondii*, MIC2, Integrin-like A domain, Yeast-two-hybird, LAMTOR1, RNaseH2B

## Abstract

**Background:**

*Toxoplasma gondii* is an obligate intracellular protozoan, causing the important zoonosis toxoplasmosis. This parasite utilizes a unique form of locomotion called gliding motility to find and invade host cells. The micronemal adhesin MIC2 plays critical roles in these processes by binding to substrates and host cell receptors using its extracellular adhesive domains. Although MIC2 is known to mediate important interactions between parasites and host cells during invasion, the specific host proteins interacting with MIC2 have not been clearly identified. In this study, we used a yeast-two-hybrid system to search for host proteins that interact with MIC2.

**Methods:**

Different adhesive domains of MIC2 were cloned into the pGBKT7 vector and expressed in fusion with the GAL4 DNA-binding domain as baits. Expression of bait proteins in yeast cells was analyzed by immuno-blotting and their autoactivation was tested *via* comparison with the pGBKT7 empty vector, which expressed the GAL4 DNA binding-domain only. To identify host proteins interacting with MIC2, a mouse cDNA library cloned into a GAL4 activation-domain expressing vector was screened by yeast-two-hybrid using the integrin-like A domain of MIC2 (residues 74–270) as bait. After initial screening and exclusion of false positive hits, positive preys were sequenced and analyzed using BLAST analysis and Gene Ontology Classifications.

**Results:**

Two host proteins that had not previously been reported to interact with *T. gondii* MIC2 were identified: they are LAMTOR1 (late endosomal/lysosomal adaptor, MAPK and mTOR activator 1) and RNaseH2B (ribonuclease H2 subunit B). Gene Ontology analysis indicated that these two proteins are associated with many cellular processes, such as lysosome maturation, signaling transduction, and RNA catabolism.

**Conclusion:**

This study is the first one to report interactions between *Toxoplasma gondii* MIC2 and two host proteins, LAMTOR1 and RNaseH2B. The data will help us to gain a better understanding of the function of MIC2 and suggest that MIC2 may play roles in modulating host signal transduction and other biological processes in addition to binding host cells.

**Electronic supplementary material:**

The online version of this article (doi:10.1186/s13071-014-0543-1) contains supplementary material, which is available to authorized users.

## Background

*Toxoplasma gondii* is an obligate intracellular protozan, which is responsible for toxoplasmosis in immunocompromised patient and livestock [1]. *T. gondii* is able to infect a wide variety of warm-blooded animals, including wildlife mammals [2], birds [3,4] and humans. The broad host range of this parasite is partially due to its successful host invasion mechanism, which is conserved among many apicomplexan parasites [5]. Invasion is a multi-step process that leads to the establishment of parasitophorous vacuoles (PV), in which the invaded parasites replicate within host cells [6]. *T. gondii* utilizes a unique mode of locomotion named “gliding motility” to get close to and actively penetrate host cells. Gliding motility is also responsible for tissue migration and local dissemination of the parasites. Powered by the actin-myosin motor complex, gliding motility also requires proteins released from the apical organelle called micronemes [7].

Previous studies have shown that micronemal proteins (MICs) of *T. gondii* have important roles in host-cell invasion [8]. The majority of MICs are adhesins, which bind to host cells during invasion. As one of the most extensively studied MICs,, MIC2 is a transmembrane adhesin that plays crucial roles during gliding motility and host-cell invasion [9-12]. Although MIC2 is not absolutely essential [13], mutants lacking MIC2 display severe growth defects and are impaired in host cell attachment, helical gliding, host-cell invasion, and virulence in mice [12]. Disruption of M2AP, a micronemal protein tightly associated with MIC2, causes partial retention of MIC2 in the secretory pathway (i.e. ER/Golgi), which leads to reduced host cell binding [14]. Similarly, disruption of TRAP (a MIC2 ortholog) in *Plasmodium berghei* results in defects in motility and host-cell invasion in sporozoites [15,16]. Once released to the parasite surface from the micronemes, MIC2 undergoes proteolytical maturation by a parasite protease called MPP2 (microneme-processing protease 2), which trims the N-terminus of MIC2 and activates its adhesive domains for substrate and receptor binding to promote gliding motility and invasion [17]. Mature MIC2 contains two different adhesive domains, a single von Willebrand factor A (vWA) integrin-like A domain (A/I) and a TSR domain containing six thrombospondin type I repeats. The A/I domain of MIC2 is able to interact with heparin, a ubiquitous glycosaminoglycan found in extracellular matrix of host cells [9]. In addition, MIC2 also interacts with intercellular adhesion molecule 1 (ICAM-1) *via* its A/I domain to facilitate the migration of *T. gondii* across polarized epithelial cells [10]. Unlike the A/I domain, the host proteins interacting with the TSR domain of MIC2 are still unclear, although thrombospondin-1 is involved in interactions with many ligands in animals and humans [18].

Many studies have shown that binding of MIC2 to host cells promotes *T. gondii* invasion, however the host receptors that mediate MIC2 binding have not been identified. Therefore, identification of more host proteins that interact with MIC2 may help us better to understand the invasion mechanism and find targets for drug discovery. In the present study, a high throughput yeast-two-hybrid screen was performed to search for host proteins that interact with *T. gondii* MIC2. Two hits, LAMTOR1 and RNaseH2B, which have not been reported previously to interact with MIC2, were identified using the A/I domain of MIC2 as bait. These two proteins are associated with many cellular processes in host cells, such as protein binding, endosome/lysosome maturation, signal transduction, RNA catabolic processes and so on. Thus, these findings indicate that MIC2 is not only involved in binding to host cells but may also affect host signal transduction and other biological processes.

## Methods

### Mice and parasites

Female KunMing mice aged 4–6 weeks were purchased from Laboratory Animals Research Centre of Hubei province, P. R. China. All the mice were raised under standard conditions and allowed access to feeding and water ad libitum. All animal experiments were performed under the approval of Laboratory Animals Research Centre of Hubei province and the ethics committee of Huazhong Agricultural University (Permit number: 4200695757).

*T. gondii* RH strain was maintained by peritoneal passage in KunMing mice. To be used in experiment for total RNA extraction, tachyzoites were harvested from peritoneal fluid of infected mice 3 ~ 4 days post infection, and were purified by filtration through CF-11 cellulose (Whatman Inc., USA).

### Bait plasmids construction

**Figure 1:**
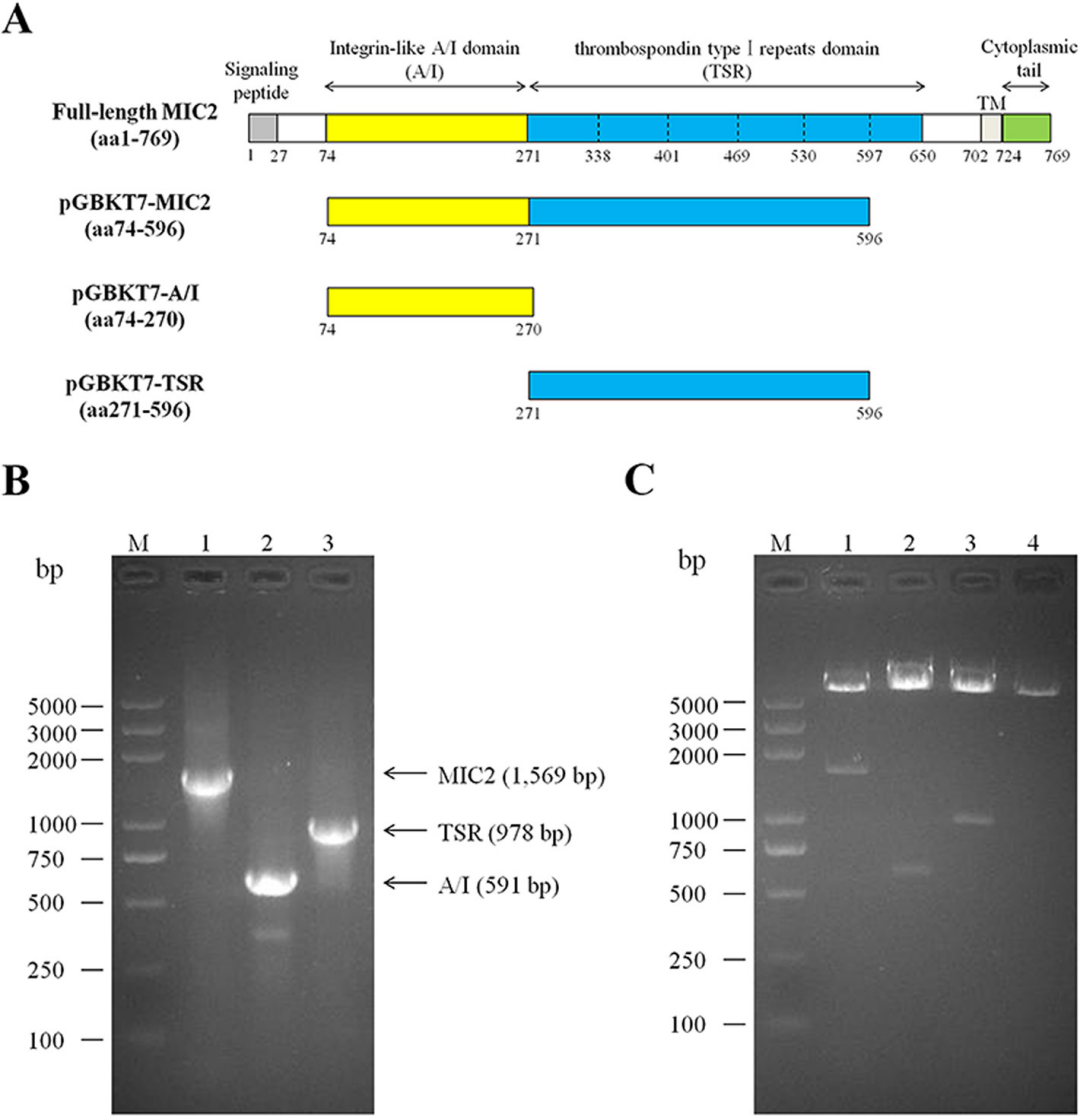
**Construction of MIC2 bait plasmids. (A)** Schematic illustration of full-length MIC2, and its different domains used as baits in the yeast-two-hybrid screen. **(B)** Agarose gel electrophoresis analysis of different fragments of MIC2 amplified from *T. gondii* cDNA. M: DNA maker; 1: MIC2 ectodomain (aa74 - 596) that lacks propeptide and the sixth TSR motif; 2: A/I domain of MIC2 (aa74 - 270); 3: the TSR domain containing five thrombospondin typeIrepeats of MIC2 (aa271 - 596). **(C)** Agarose gel electrophoresis analysis of constructed bait plasmids digested with NdeI and SalI. M: DNA maker; 1: pGBKT7-MIC2; 2: pGBKT7-A/I; 3: pGBKT7-TSR; 4: pGBKT7 vector.

Total RNA from purified *T. gondii* tachyzoites was extracted using TRIzol Reagent according to manufacturer’s instructions (Invitrogen, USA). One microgram of total RNA was reverse-transcribed into cDNA using the ReverTra Ace reverse transcription kit according to manufacturer’s instructions (TOYOBO, Japan). The bait plasmids were constructed by cloning different fragments of MIC2 (TGGT1_210780, http://toxodb.org/toxo/) into the pGBKT7 vector. These MIC2 fragments include (see Figure 1A for illustration): the 1,569 bp fragment encoding a 523-residue (from aa74 to aa596) peptide of MIC2 ectodomain (to give pGBKT7-MIC2); the 591 bp fragment encoding the A/I domain (197 amino acid residues, from aa74 to aa270) (to give pGBKT7-A/I); and the 978 bp fragment encoding the TSR domain (326 amino acid residues, from aa271 to aa596) (to give pGBKT7-TSR). These fragments were PCR amplified from cDNA using corresponding primers listed in Table 1 and cloned into pGBKT7 between NdeI and SalI sites.

**Table 1:** **Primers used to amplify different domains of MIC2 from cDNA of** ***T. gondii*** **tachyzoites**

**Primer name**	**Sequence** ^**1**^	**Restriction enzyme sites**
pGBKT7-MIC2-F	GC *CATATG* CAGCTGGACATTTGCTTTC	*NdeI*
pGBKT7-MIC2-R	TA *GTCGAC* GACAAGCAGGATACGAACATG	*SalI*
pGBKT7-A/I-R	TA *GTCGAC* TGGCATCCTGGGGGAGTGTCTT	*SalI*
pGBKT7-TSR-F	GC *CATATG* GCCATTTGCTCGGATTGGTC	*NdeI*

^1^Restriction enzyme sites were presented in italics. Primers were used in the following combinations for PCR amplifications: pGKBT7-MIC2-F and pGKBT7-MIC2-R were used to amplify MIC2 ectodomain (aa74 - 596); pGBKT7-MIC2-F and pGKBT7-A/I-R were used to amplify the A/I domain (aa74 - 270); and pGKBT7-TSR-F and pGKBT7-MIC2-R were used to amplify the TSR domain (aa271 - 596).

### MIC2 Baits expression in yeast cells

To check the expression of MIC2 baits in yeast cells, each bait plasmid was transformed into *Saccharomyces cerevisiae* strain Y2HGold (*MATa, trp1-901, leu2-3, 112, ura3-52, his3-200, gal4Δ, gal80Δ, LYS2 : : GAL1*_*UAS*_*–Gal1*_*TATA*_*–His3, GAL2*_*UAS*_*–Gal2*_*TATA*_*–Ade2 URA3 : : MEL1*_*UAS*_*–Mel1*_*TATA*_*AUR1-C MEL1*) using the lithium acetate method according to the User’s Manual of Yeastmaker™ Yeast Transformation System 2 (Cat. No. 630439, Clontech, USA). Transformants were selected on plates containing the minimal yeast medium without tryptophan (SD/-Trp) for 3 ~ 5 days. Subsequently one colony from the SD/-Trp plate was inoculated into SD/-Trp broth and grown until the OD_600_ reached 0.6. Then total proteins were extracted from pelleted cells by the Urea/SDS method [19]. Extracted proteins were then separated on 12% SDS-PAGE gels and transferred electrophoretically to PVDF membrane (Millipore., USA) for Western blotting analysis. The MIC2 baits were c-Myc tagged and were detected with a mouse anti-Myc antibody (Medical and Biological Laboratories Co., Ltd., Japan) followed by peroxidase-conjugated goat anti-mouse secondary antibody (Beyotime., China). The blot was developed using the direct ECL chemiluminescent method (Thermo scientific, USA).

### Autoactivation test

To test autoactivation of the bait proteins, each MIC2 bait plasmid was transformed into the yeast strain Y2HGold as above. Transformants were then grown on plates containing the minimal yeast medium without tryptophan (SD/-Trp), or SD/-Trp supplemented with 40 ug/ml X-α-Gal (SD/-Trp/X), or SD/-Trp supplemented with 40 ug/ml X-α-Gal and 125 ng/ml Aureobasidin A (SD/-Trp/X/A). Lack of autoactivation was indicated by white colonies on SD/-Trp and SD/-Trp/X plates and absence of colony growth on SD/-Trp/X/A plates. The bait that did not possess autoactivation activity was used in the yeast-two-hybrid screen.

### Yeast-two-hybrid screen

All yeast strains, reagents and methods for yeast-two-hybrid assays were from the Matchmaker™ Gold Yeast Two-Hybrid System (Cat. No. 630489, Clontech, USA). To screen host proteins that interact with the A/I domain of MIC2, *S.cerevisiae* Y187 cells (*MATα, ura3-52, his3-200, ade2-101, trp1-901, leu2-3, 112, gal4Δ, gal80Δ, met–, URA3 : : GAL1*_*UAS*_*–Gal1*_*TATA*_*–LacZ, MEL1*) containing the Mate & Plate™ Universal Mouse (Normalized) cDNA library (Cat. No. 630482, Clontech., USA) cloned into the pGADT7-RecAB vector were used to mate with the Y2HGold cells transformed with the bait plasmid for 24 h at 30°C. The mated culture was then spread onto SD/-Leu/-Trp plates supplemented with 40 ug/ml X-α-Gal and 125 ng/ml Aureobasidin A (DDO/X/A). Blue colonies (which were potential positive hits) were restreaked onto higher stringency SD/-Ade/-His/-Leu/-Trp plates supplemented with 40 ug/ml X-α-Gal and 125 ng/ml Aureobasidin A (QDO/X/A). The prey plasmids in positive clones were isolated using TIANprep yeast plasmid DNA kit (TIANGEN, China) and purified by transformation of *E. coli* DH5α cells followed by selection on LB/Amp plates. To estimate the sizes of the specific inserts on positive prey plasmids, PCR analysis was carried out using the primers provided by Matchmaker™ AD LD-Insert Screening Amplimer Set (Cat. No. 630433, Clontech, USA). To exclude false positive hits, each putatively positive prey plasmid from the initial screen was co-transformed into Y2HGold strain with the bait plasmid. Transformants were then spread onto QDO/X/A plates to test for interactions. True positive hits were indicated by blue colonies under these conditions.

### Positive prey analysis

The positive prey plasmids were further characterized by determining the DNA sequences of the inserts using Sanger sequencing. The sequencing results of positive hits were used in BLAST search to identify the corresponding mouse genes. Gene Ontology Classifications of identified genes were analyzed using the Mouse Genome Informatics database (http://www.informatics.jax.org/).

## Results

### Construction of bait plasmids expressing different domains of MIC2

The coding sequence of MIC2 ectodomain, which lacks propeptide and the sixth (last) TSR motif, was amplified from tachyzoites cDNA (Figure 1B lane 1), and cloned into pGBKT7 between the NdeI and SalI sites to be expressed in frame with the GAL4 DNA-binding domain. We also constructed two additional baits which express either the A/I domain or the TSR domain of MIC2, respectively. The coding sequences of the two domains were amplified from tachyzoites cDNA (Figure 1B lane 2 and 3) and cloned into pGBKT7 as above. Successful construction of these plasmids was confirmed by restriction enzyme digestion analysis (Figure 1C).

### Expression and auto-activation test of MIC2 baits in yeast cells

**Figure 2:**
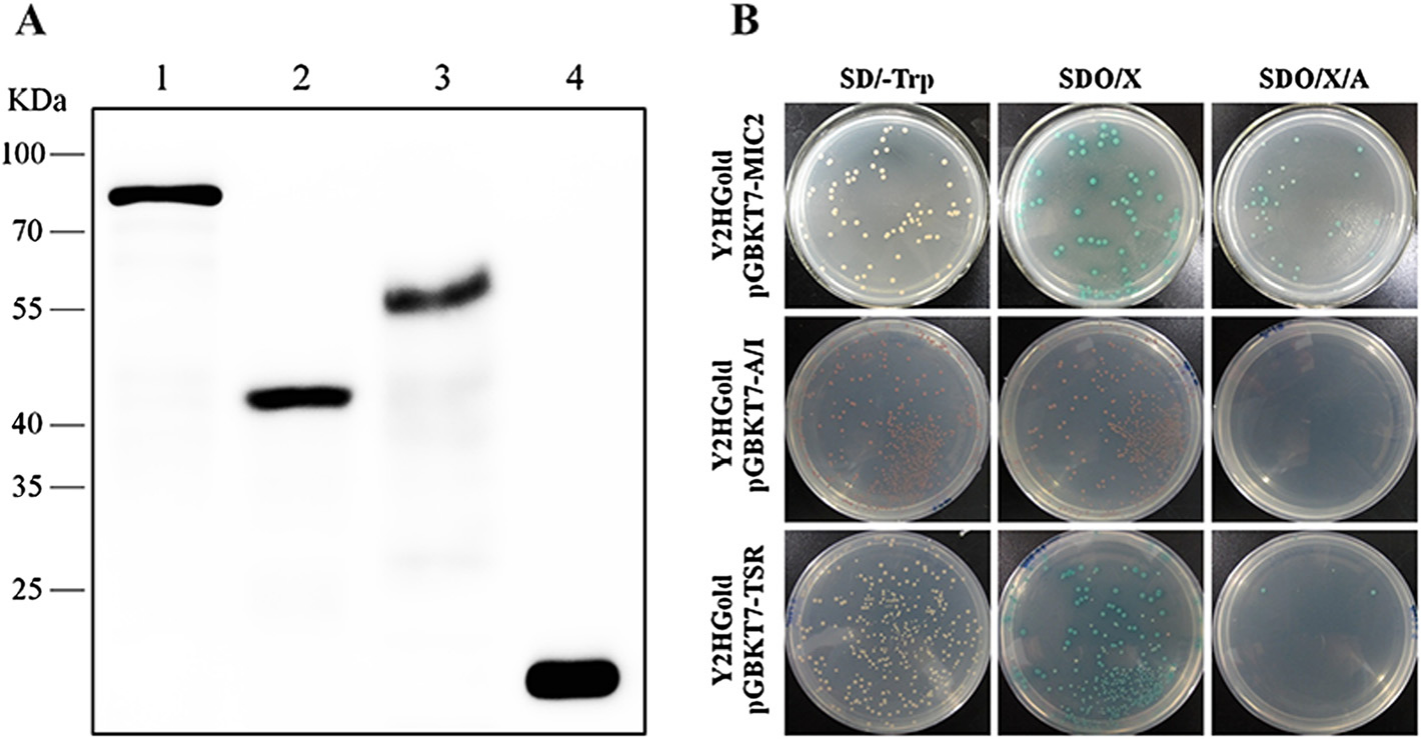
**Expression and autoactivation of MIC2 baits in yeast cells. (A)** Western blotting analysis on lysate of the yeast strain Y2HGold containing the following MIC2 bait plasmids. 1: pGBKT7-MIC2; 2: pGBKT7-A/I; 3: pGBKT7-TSR; 4: pGBKT7. **(B)** Determination of the auto-activation activity of different MIC2 baits in yeast cells.

Prior to yeast-two-hybrid screening, expression of the baits in yeast *Saccharomyces cerevisiae* was examined. Total proteins of the yeast strain Y2HGold pre-transformed with each corresponding bait plasmid (pGBKT7-MIC2, or pGBKT7-A/I, or pGBKT7-TSR) were extracted and analyzed by Western blotting using the Myc-tag antibody. The results indicated that all three bait proteins were expressed and easily detected by immuno-blotting. The MIC2 ectodomain bait fusion was detected to have a molecular weight (MW) around 78 kDa, which is consistent with its calculated size (Figure 2A lane 1). Similarly, the A/I domain and the TSR domain fusions were detected around 43 kDa and 56 kDa, respectively (Figure 2A lane 2 and 3). As a control, the pGBKT7 empty vector transformed Y2HGold cells expressed the GAL4 DNA-binding domain that was 21 kDa in size (Figure 2A lane 4).

To test the autoactivation activity of these bait proteins in yeast cells, each bait plasmid was individually transformed into Y2HGold cells and subsequently the transformants were grown on minimal yeast media plates (i.e. SD/-Trp, SD/-Trp/X and SD/-Trp/X/A) to test autoactivation (Figure 2B). Autoactivation activities from the baits would enable the expression of reporter genes and lead to blue colonies on SD/-Trp/X plates and SD/-Trp/X/A plates. By doing so, we detected robust autoactivation activity for MIC2 ectodomain (A/I domain + TSR domain) and the TSR domain of MIC2 (Figure 2B). No autoactivation activity was detected from the A/I domain (Figure 2B), suggesting that the TSR domain was responsible for the autoactivation activity of the MIC2 ectodomain. According to these results, only the bait containing the A/I domain of MIC2 was used in the yeast-two-hybrid screen described below.

### Yeast-two-hybrid screen against a mouse cDNA library using the A/I domain of MIC2 as bait

**Figure 3:**
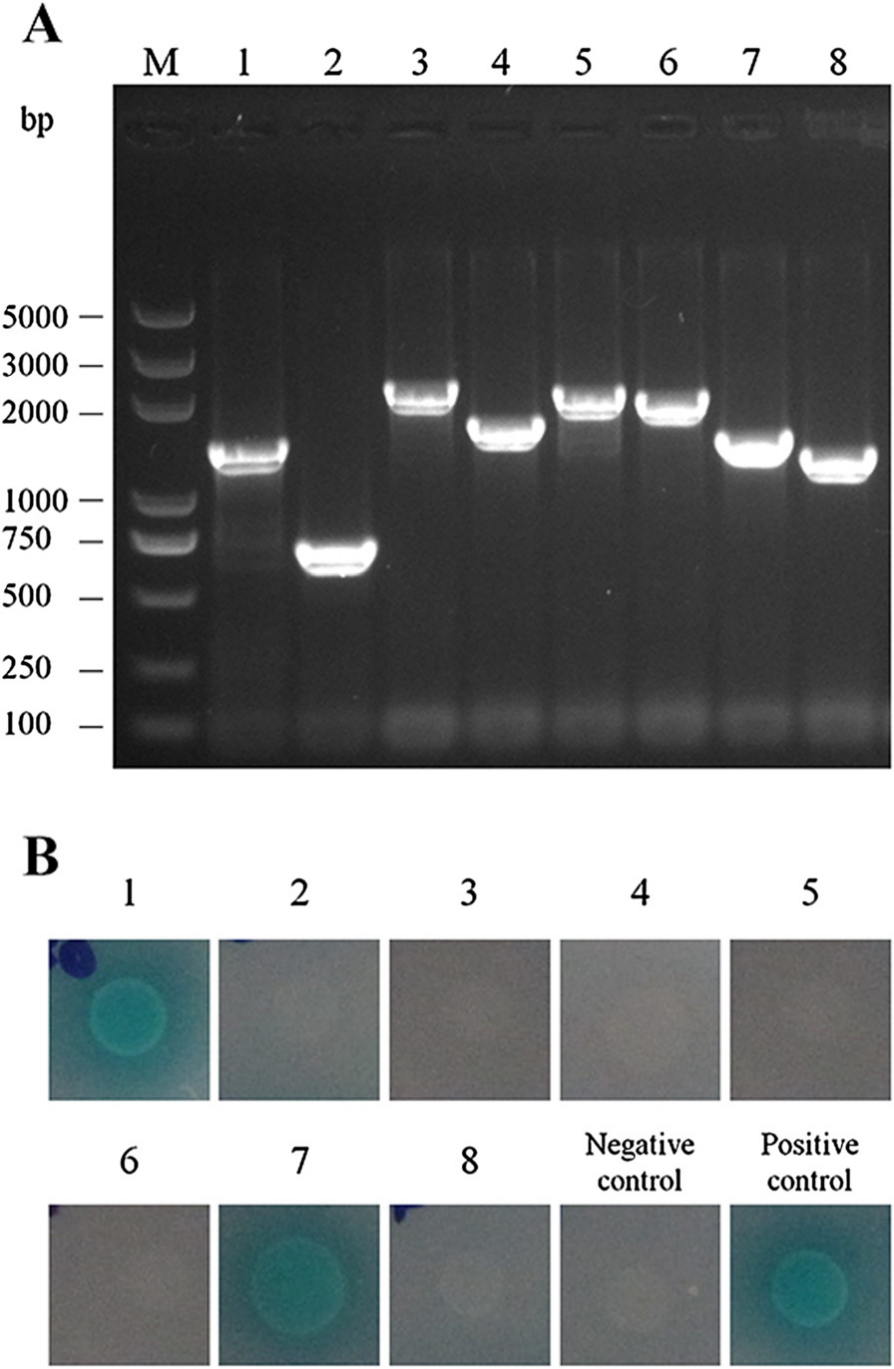
**Analysis of putatively positive hits obtained from the yeast-two hybrid screen. (A)** Agarose gel electrophoresis analysis of PCR products obtained from amplification of the inserts on putatively positive prey plasmids. M: DNA maker; Lane 1–8: PCR amplification products of the inserts on the eight putatively positive hits. **(B)** Confirmation of putative hits. Y2HGold cells were co-transformed with pGBKT7-A/I and each of the eight putatively positive prey plasmids (number 1 to 8) and plated on QDO/X/A plates; positive interaction was indicated by the presence of blue colonies. Co-transformation with pGADT7-T and pGBKT7-Lam was used as a negative control, whereas co-transformation with pGADT7-T and pGBKT7-53 was used as a positive control.

To search for host proteins that interact with the A/I domain of MIC2 by yeast-two-hybrid, Y2HGold cells harboring the pGBKT7-A/I plasmid was used to mate with Y187 cells containing the Mate & Plate™ Universal Mouse (Normalized) cDNA library. After mating and growth on different selection plates, the mating efficiency was calculated to be 9.68% (data not shown). Based on this efficiency, we screened about 10^9^ viable diploid yeast cells. Among the >200 large colonies (2 mm – 3 mm) grown on DDO/X/A plates, twenty of them were blue, a phonotype that positive hits should have. Subsequently, these twenty blue clones were restreaked onto higher stringency QDO/X/A selection plates. Eight out of the twenty still gave blue colonies, indicating that they were likely to be positive hits. The prey plasmids were then isolated from these putatively positive clones and rescued through transformation of *E.coli* DH5α cells. The specific insert on each prey plasmid was PCR amplified using primers originated from the vector backbones and analyzed by gel electrophoresis (Figure 3A). To eliminate false positive hits and retest the specificity of interaction, each of the eight prey plasmids was co-transformed with pGBKT7-A/I into Y2HGold cells and the co-transformants were tested on the QDO/X/A plates. The results indicated that two of the eight preys still showed positive interaction with the A/I bait but the other six did not (Figure 3B). To further check the interaction specificity of the two hits, we co-transformed each of the two prey plasmids with pGKBT7 empty vector into Y2HGold yeast cells and tested the growth and color of transformants on QDO/X/A plates. In both cases, we did not observe any colony growth on QDO/X/A plates, suggesting that both hits had specific interactions with the A/I domain of MIC2 (data not shown). Thus, two host proteins were identified to interact with the A/I domain of MIC2 and were further analyzed as below.

### Sequencing and analysis of positive prey

To investigate the identity of host proteins in our positive hits, we sequenced the two prey plasmids using the T7 primer, which was located upstream of the inserts on the prey plasmids. The sequencing results (Additional file 1) were then used to search the mouse genome using the BLAST program (http://blast.ncbi.nlm.nih.gov/Blast.cgi). The results showed that the two inserts had high similarity (99% ~ 100%) to two known genes of *Mus musculus*: the late endosomal/lysosomal adaptor MAPK and mTOR activator 1 (*Lamtor 1*, NM_025605) and the ribonuclease H2 subunit B (*Rnaseh2b*, NM_026001). Sequencing also showed that the *Lamtor 1* containing hit had the full length *Lamtor 1* on the plasmid in frame with the GAL4 DNA-binding domain, whereas the *Rnaseh2b* containing hit only had the C-terminus of the protein (from residue 111 to residue 308) (Additional file 1). These two proteins had not been reported to interact with MIC2 before. Gene ontology classifications and putative functions of these two proteins are shown in Table 2. The results indicated that LAMTOR1 functions as an adaptor involved in host signal transducation and RNaseH2B possesses ribonuclease activity during many cellular processes. Taken together, we discovered the interactions between MIC2 and two mouse proteins (LAMTOR1 and RNaseH2B), which may help understanding the cellular functions of MIC2 in greater detail.

**Table 2:** **The BLAST results and GO analysis of positive hits from the yeast-two-hybrid screen**

**Prey protein name**	**Genbank accession No.**	**Gene ontology classifications**	
		**Molecular function**	**Biological process**
Late endosomal/lysosomal adaptor MAPK and mTOR activatr 1 (**LAMTOR1**)	NM_025605	Guanyl-nucleotide exchange factor activity	Cellular protein localization
		Protein binding	Cellular response to amino acid stimulus
		Protein complex scaffold	Endosome/Lysosome localization and organization
			Positive regulation of MAPK cascade
			Positive regulation of TOR signaling
			Regulation of cholesterol homeostasis
			Regulation of GTPase activity
			Regulation of receptor recycling
Ribonuclease H2 subunit B (**RNaseH2B**)	NM_026001	RNA-DNA hybrid ribonuclease activity	In utero embryonic development
			Negative regulation of gene expression
			Positive regulation of fibroblast proliferation
			Regulation of DNA damage checkpoint
			Regulation of G2/M transition of mitotic cell cycle
			Ribonucleotide metabolic process
			RNA catabolic process

## Discussion

Although previous studies have demonstrated the interaction between MIC2 and host receptors during invasion [9,10], the specific host receptors involved in this interaction are still not clear. In order to identify host proteins that interact with MIC2, we took a high throughput yeast-two-hybrid screen strategy to search for mouse proteins that interact with MIC2. Three bait plasmids that expressed different parts of the MIC2 ectodomain were initially designed for the yeast-two-hybrid screen. Recent structural studies on *Toxoplasma gondii* MIC2 suggested that the MIC2 A/I domain can exist in both open and closed conformations [20]. Conversion of the closed conformation to the open conformation may occur after the cleavage of propeptide. Previous studies showed that open integrin I domains have higher receptor binding affinities than closed ones [21,22]. Thus, we thought that the MIC2 ectodomain lacking the propeptide would be a good bait to screen for binding partners, since it would be in an open conformation. In addition, the sixth TSR motif of the MIC2 ectodomain is only partially conserved and was found to be involved in M2AP binding [20]. Therefore, the sixth TSR motif was also excluded from our baits. In total, we constructed three baits: the A/I domain +5 TSR domains, the A/I domain alone and the 5 TSR domains alone.

Prior to the yeast-two hybrid screen, we tested the auto-activation activity of the three baits and found that the baits containing the 5 TSR domains showed auto-activation in yeast. This implies that the TSR domains may have eukaryotic transcriptional activation activities, which contributed to the auto-activation of MIC2 baits in yeast cells. To date, there are no data reporting interactions between the TSR domains of MIC2 and host proteins or carbohydrates, although its adhesive properties and motif conservation have been found in other species of apicomplexan parasites [23]. Thus, the TSR domains may play other roles for MIC2 function. Unlike the TSR domains, the A/I domain does not possess any detectable autoactivation activity and is expressed well in yeast cells. Therefore, we used the A/I domain of MIC2 as bait for our yeast-two hybrid screen.

Two novel mouse proteins, LAMTOR1 and RNaseH2B, were identified in our screen. This is the first study reporting interactions between MIC2 and these two host proteins. LAMTOR1 (late endosomal/lysosomal adaptor MAPK and mTOR activator 1), also called p18, is a membrane adaptor protein that localizes exclusively to the surface of lysosomes and late endosomes/lysosomes [24]. It serves as an anchor for the p14-MP1 complex that is involved in the late stages of lysosomal maturation, especially late endosome-lysosome fusion. LAMTOR1 and the LAMTOR1-p14-MP1 complex is highly conserved from yeast to humans [25]. Knockout of LAMTOR1 in mice leads to severe defects in endosome/lysosome organization and embryonic lethality during early developmental stages [24]. LAMTOR1 deficient cells show aberrant distribution of lysosomes through perinuclear compartments, because of the exclusion of the p14-MP1 complex from the late endosomes. Recent studies show that the LAMTOR1-p14-MP1 complex is essential for regulating the activity of the mammalian Target Of Rapamycin Complex 1 (mTORC1) on lysosomes [26]. Conditional ablation of LAMTOR1 in mouse epidermis suggests that the LAMTOR1 related complex is involved in promoting lysosome-mediated degradation of cellular components, and is also required for regulating the formation of autolysosome [27]. Furthermore, LAMTOR1 also plays a potential role in p53-dependent apoptosis by regulating lysosomal activation [28]. Although still needs to be examined *in vivo*, our finding that MIC2 interacts with LAMTOR1 suggests that *T. gondii* infection may affect the above LAMTOR1-dependent host activities through MIC2- LAMTOR1 interaction.

The other mouse protein came out of our screen is RNaseH2B, a subunit of the ribonuclease (RNase) H2 enzyme complex, which shares high sequence homology in humans and mice [29]. The eukaryotic RNase H2 enzyme carries most of the RNase H activity in mammalian cells, which not only hydrolyzes the RNA strand of RNA/DNA hybrids but also recognizes single ribonucleotides in a DNA duplex and cleaves the 5’-phosphodiester bond of the ribonucleotide [29]. The RNase H2 complex consists of RNaseH2B and two other subunits: RNaseH2A and RNaseH2C. In this enzyme complex, the RNaseH2A subunit provides the main catalytic activity, whereas the RNaseH2B and RNaseH2C subunits are possibly involved in interactions with other proteins [30-32]. RNaseH2B contains a PIP box at the C-terminus, which guides the interaction between RNase H2 enzyme complex and the proliferating cell nuclear antigen (PCNA), a protein involved in DNA replication [33]. Mutations in the genes encoding RNaseH2B subunits cause the Aicardi-Goutières syndrome (AGS), a neurological inflammatory disorder found in children [34]. This early-onset of neuroinflammation also has immunological similarities to the autoimmune disease systemic lupus erythematosus [35]. These observations suggest that RNaseH2B also plays crucial roles in the development of the nervous and immune systems. In addition, ablation of RNaseH2B in mice causes early embryonic lethality due to elevated DNA damage and reduced cell proliferation during gastrulation [36]. Given the multiple roles of RNaseH2B in host cells, *Toxoplasma* infection may modulate these RNaseH2B dependent processes through MIC2-RNaseH2B interaction, which deserves further investigation.

Although we have found two host proteins to interact with MIC2 from the yeast-two-hybrid screen, there are probably other host proteins that also interact with MIC2 but were not found in our screen, due to the limitations of this technology. For example, in this screen we did not find ICAM1, a protein known to interact with the A/I domain of MIC2. Possible explanations for this include: first, ICAM1 is a secretory protein associated with the membrane, which may be difficult to be localized to yeast nucleus, making it hard to be identified in the two-hybrid screen. Secondly, this Universal Mouse (Normalized) cDNA library may not contain any clones that express ICAM1 in the correct manner. Finally, previous studies have shown that multimerization of the A/I domain is required for host cell binding [9]. However, this may not occur inside yeast cells. Given these limitations, it is possible that there are other MIC2 binding partners that were missed from our screen. Further efforts should be taken to identify these other interaction partners in the future to gain more insights into the function of MIC2.

## Conclusion

Two host proteins, LAMTOR1 and RNaseH2B, which have not been previously reported to associate with *Toxoplasma gondii* infection, were identified by yeast-two-hybrid screen to interact with MIC2 using the A/I domain of MIC2 as bait. These two host proteins are involved in many cellular processes, such as protein binding for signal transduction, lysosome maturation, RNA catabolism and DNA replication. Although the specific interactions between MIC2 and these two host proteins *in vivo* and the functional consequences of such interactions still need be confirmed further, the results from this study imply that micronemal proteins are not only involved in host cell binding but may also play roles in modulating host cell signaling and other biological processes during *Toxoplasma gondii* infection.

## Additional file

Additional file 1: Figure S1. Sequencing results of the inserted fragments on positive hits. Sanger sequencing was carried out using the T7 sequencing primer. The sequences of the inserts obtained from sequencing reactions and the position of insertion on the vector are shown. (A) LAMTOR1 containing hit; (B) RnasH2B containing hit. The amino acid sequences of the corresponding proteins were underlined by dotted lines.
