# Supplementary figures and images for: Identification of host proteins interacting with the integrin-like A domain of Toxoplasma gondii micronemal protein MIC2 by yeast-two-hybrid screening
Source: Parasit Vectors. 2014 Nov 26;7:543. doi: 10.1186/s13071-014-0543-1 (PMC4258286; doi:10.1186/s13071-014-0543-1)

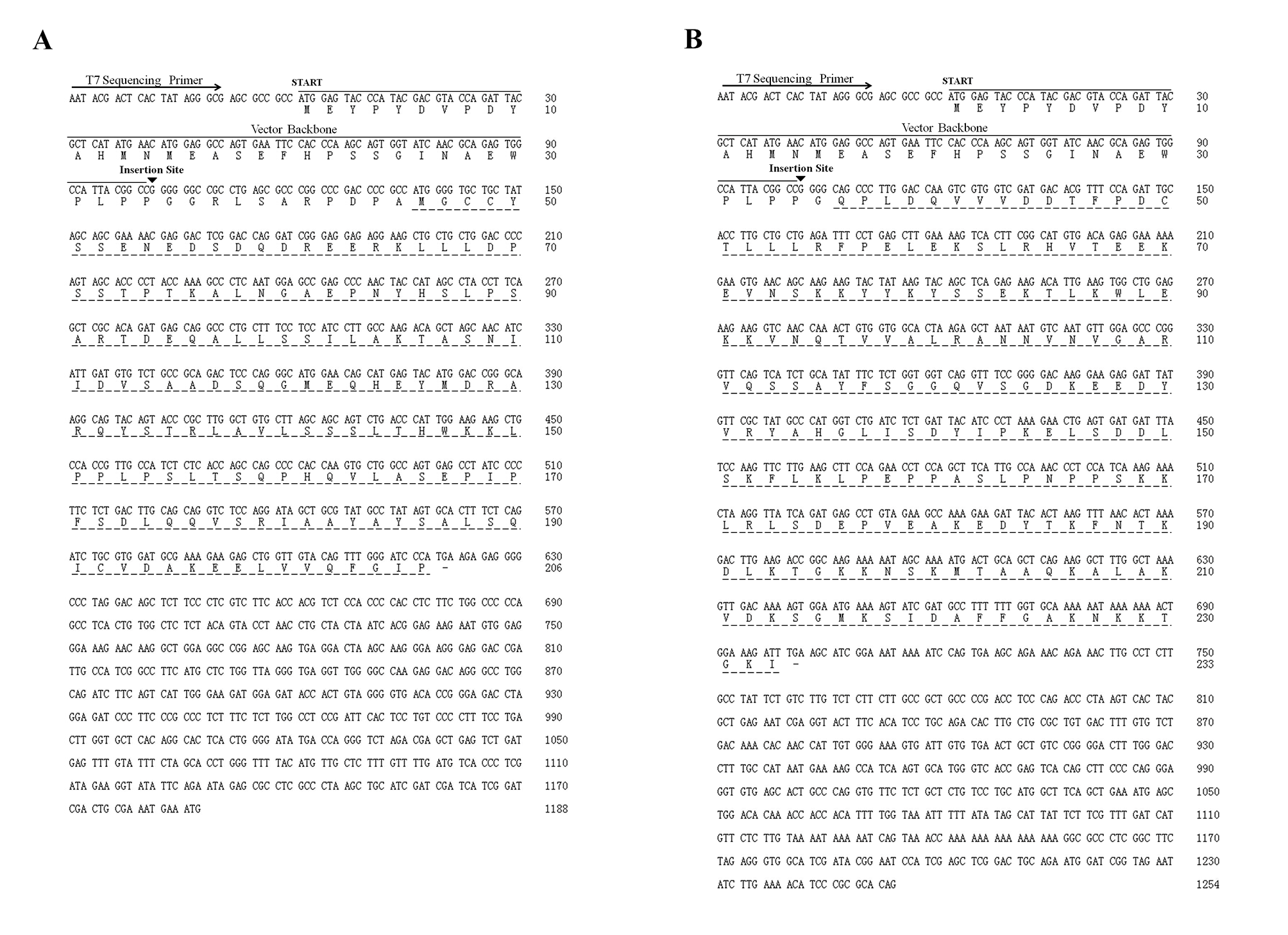

Supplement: Additional file 1: Figure S1. — Sequencing results of the inserted fragments on positive hits. Sanger sequencing was carried out using the T7 sequencing primer. The sequences of the inserts obtained from sequencing reactions and the position of insertion on the vector are shown. (A) LAMTOR1 containing hit; (B) RnasH2B containing hit. The amino acid sequences of the corresponding proteins were underlined by dotted lines. [file 13071_2014_543_MOESM1_ESM.tiff]
